# Mobile Detection and Alarming Systems for Hazardous Gases and Volatile Chemicals in Laboratories and Industrial Locations

**DOI:** 10.3390/s21238128

**Published:** 2021-12-04

**Authors:** Mohammed Faeik Ruzaij Al-Okby, Sebastian Neubert, Thomas Roddelkopf, Kerstin Thurow

**Affiliations:** 1 Technical Institute of Babylon, Al-Furat Al-Awsat Technical University (ATU), Kufa 54003, Iraq; 2 Center for Life Science Automation (Celisca), University of Rostock, 18119 Rostock, Germany; kerstin.thurow@celisca.de; 3 Institute of Automation, University of Rostock, 18119 Rostock, Germany; sebastian.neubert@celisca.de (S.N.); Thomas.roddelkopf@celisca.de (T.R.)

**Keywords:** hazardous gases, toxic gases, gas sensor, safety system, volatile organic materials (VOCs), alarming system, internet of things (IoT), wireless sensor networks (WSNs)

## Abstract

The leakage of hazardous gases and chemical vapors is considered one of the dangerous accidents that can occur in laboratories, workshops, warehouses, and industrial sites that use or store these substances. The early detection and alarming of hazardous gases and volatile chemicals are significant to keep the safety conditions for the people and life forms who are work in and live around these places. In this paper, we investigate the available mobile detection and alarming systems for toxic, hazardous gases and volatile chemicals, especially in the laboratory environment. We included papers from January 2010 to August 2021 which may have the newest used sensors technologies and system components. We identified (236) papers from Clarivate Web of Science (WoS), IEEE, ACM Library, Scopus, and PubMed. Paper selection has been done based on a fast screening of the title and abstract, then a full-text reading was applied to filter the selected papers that resulted in (42) eligible papers. The main goal of this work is to discuss the available mobile hazardous gas detection and alarming systems based on several technical details such as the used gas detection technology (simple element, integrated, smart, etc.), sensor manufacturing technology (catalytic bead, MEMS, MOX, etc.) the sensor specifications (warm-up time, lifetime, response time, precision, etc.), processor type (microprocessor, microcontroller, PLC, etc.), and type of the used communication technology (Bluetooth/BLE, Wi-Fi/RF, ZigBee/XBee, LoRa, etc.). In this review, attention will be focused on the improvement of the detection and alarming system of hazardous gases with the latest invention in sensors, processors, communication, and battery technologies.

## 1. Introduction

**Figure 1:**
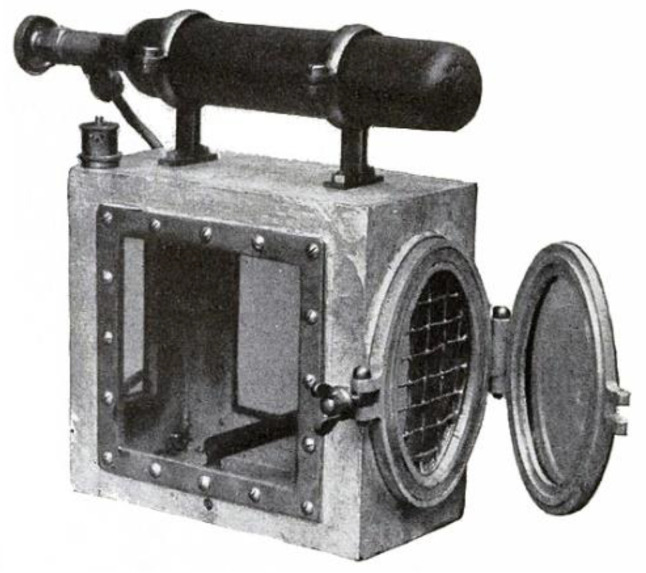
Canaries as toxic gas detectors [4].

The term hazardous gases refer to the toxic/reactive behavior, and it can be conformed to one of the following definitions: harmful to living subjects, dangerously reactive, acutely toxic, flammable, corrosive, or oxidizing gases [1,2,3]. The urgent need for detection and warning devices of odorless, harmful, and toxic gases and vapors in places exposed to this, prompted the use of primitive methods that may be harsh to protect the lives of the public in these places. Until the eighties of the previous century, miners used canaries kept in a special glass cage to detect the presence of toxic gases (especially carbon monoxide) when entering the mines (see Figure 1). Since the effect of harmful gases is greater and faster on the canaries than humans, workers were alerted to any possible danger by the ‘bird sensors’. The system included an oxygen chamber to save the bird’s life after facing the toxic gases [4].

Recently, the considerable development in laboratory infrastructures and industries increases the use of robots, drones, quadcopters, and automated transportation systems used besides humans in chemical, biological, and biotechnological processes. Moreover, numerous hazardous gases and chemicals vapors in urban places, such as laboratories in universities, research centers of companies, and big factories which may have warehouses for chemical gases/compounds and can be faced with accidental leakages of hazardous gases or vapors. Thus, sensitive systems to detect and alarm any dangerous leakage that may affect the laboratory staff or the environment, in general, are required. 

Many hazardous and toxic gases require special conditions for their handling since they can cause a series of health hazards such as death or permanent injury. Typical inorganic compounds/gases include ammonia, arsine, carbon monoxide, hydrogen bromide, hydrogen chloride, hydrogen cyanide, hydrogen sulfide, nitric oxide, nitrogen dioxide, ozone, phosgene, phosphine, or sulfur dioxide. Of increasing interest are organic compounds such as saturated, unsaturated, and aromatic hydrocarbons, organochlorine compounds, organic amines, organic silanes, alcohols, esters, aldehydes, and ketones. Compounds with a very high vapor pressure are summarized under the term volatile organic compounds (VOCs) and represent the greatest danger to humans due to their easy evaporation [5,6,7]. The National Fire Protection Association (NFPA) created a numerical system from 0–4 for classifying the gas hazard rating based on OSHA (Occupational Safety and Health Administration) hazard communication standard where 0–4 refer to minimal, slight, moderate, serious, and severe hazard respectively [8]. 

**Figure 2:**
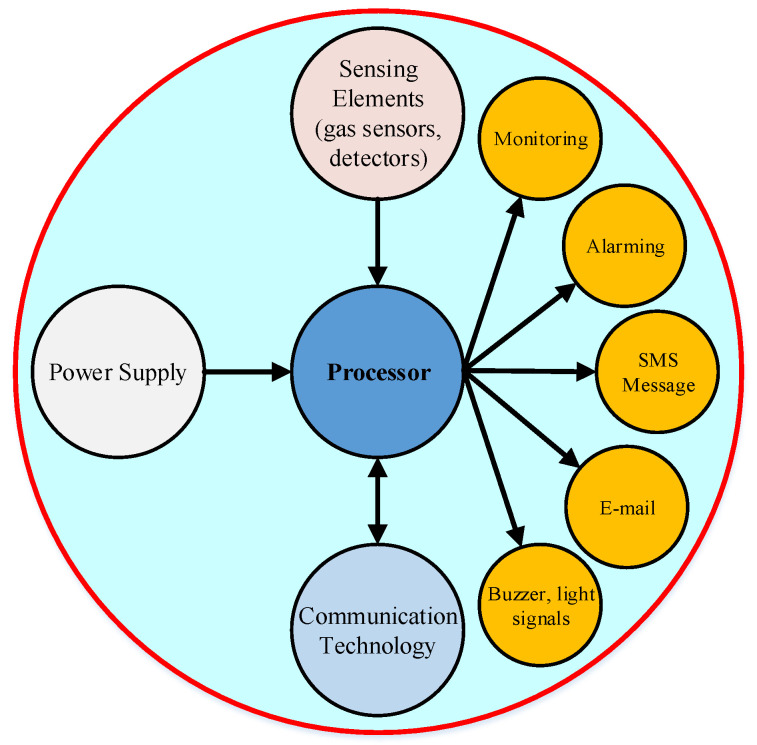
Basic block diagram of mobile detecting and alarming system.

Suitable sensor systems have to be developed to enable an easy, reproducible, and reliable detection of possible chemical hazards in the working environment. There are several main parts for a compact mobile detecting and alarming system (see Figure 2) which such as:

### 1.1. Sensing Element (Gas Sensor)

The sensing elements represent the most important part that implements the target goal of detecting toxic and hazardous gases. Several technologies have been used in the manufacturing of gas sensors and based on that different sensor detecting procedures can be used. Each type of gas sensor has the characteristics that make it a better fit for specific environments and gases. In general, five main sensing principles are used for the detection: 

#### 1.1.1. Catalytic Sensors

This type of gas sensor measures the temperature difference between two beads (inert and with catalytic substance) in case of different heat responses. The catalytic bead method is mainly useful for the detection of combustible gases such as natural gas, methane, butane, propane, or hydrogen. Especially in the detection of hydrocarbons of the lower explosive level (LEL) or hydrogen (H_2_) this method is used to monitor explosion limits. Catalytic bead sensors are inexpensive and robust and can easily be calibrated due to the linear sensor reaction depending on the gas concentration. The disadvantages of the old bulky catalytic sensors are the low sensitivity in the percentage ranges as well as the low selectivity since this type of sensor registers every gas that burns on the catalyst surface of the pellistor and causes a measurable heat release. In addition, the sensors consume a significant amount of electrical power due to the required heating. The new MEMS-based catalytic sensors show a better selectivity with high sensitivity and very low power consumption [9,10,11]. 

#### 1.1.2. Electrochemical Sensors

The electrochemical gas sensor (also electrochemical cells or EC sensors) uses oxidization processes to generate an electric current in electrodes. The generated current will be amplified and measured by an external circuit. The typical electrochemical gas sensor consists of three parts. The first is the gas chamber, which is located on the upper layer of the sensor to let the gases pass through the sensor. The second part is the electrochemical cell, which is located at the middle layer of the sensor. The electrochemical reaction takes place inside the electrochemical cell, which includes the electrodes (“working” electrode, a “counter” electrode, and a “reference” electrode) embedded in an electrolyte solution. The third part is the electrolyte reservoir, which is located in the lower layer of the sensor and is responsible for the change in the electrolyte concentration equilibrium. The working principle of the electrochemical sensor is based on gas diffusion. When the gas passes through the gas chamber and reaches the working electrode, an electrochemical reaction will occur depending on the gas type. The reaction causes the movement of electrons from the working electrode to the counter electrode, which can be measured by an external circuit. The value of the generated current depends on the concentration of the target gases. The electrochemical sensors have attractive specifications such as high sensitivity, high selectivity, low-power consumption, and low-cost production. The main drawback is the high sensitivity to relative humidity, temperature, and high gas concentrations, which can cause electrode poisoning. Chemical sensors also show a high-temperature dependence, resulting in offsets or influencing the sensor response times [12,13,14,15,16,17]. 

#### 1.1.3. Photoionization Sensors

This sensor type uses the light ionization characteristic of gases to generate electric currents from positive and negative ions. This principle is primarily used for the detection of harmful VOCs in the environmental air. Modern PID solutions are already capable to measure concentrations of organic compounds around 1–10 ppb and have typical response times of a few seconds [10,11,12,13].

#### 1.1.4. Infrared Sensors/Optical Sensors

The working principle of this type is based on the comparison between the actively absorbed wavelength and the reference stored wavelength (not absorbed by gases). The NDIR method is very robust and cost-effective for measuring gases with a medium resolution. Typical response times are in the region of about 20 s. Infrared and optical sensors allow small constructions which enable portable solutions. Additional reference detectors can be used to recognize many disturbances and to avoid false detections. The method can only be used for gases that absorb infrared light at known wavelengths [10,18,19,20].

#### 1.1.5. MOX Sensors/Chemical Sensors

Metal oxide semiconductor sensors use a resistive principle, there the gas or gas mixture to be measured directly influences the conductivity of a gas-sensitive sensor layer. This change in resistance serves as a measured variable. Besides metal oxide-based sensors also organic phthalocyanines or conductive polymers, metal alloys, and transition metal dichalcogenides can be used as a sensing layer. MOX sensors show high sensitivity and can thus be used for the determination of small concentrations. They show a non-linear response to the gases. In addition, they only have low selectivity. Thus, their use is limited to the determination of known compounds [18,21,22].

### 1.2. Processor

The processor represents the brain of the system; it can be a microcontroller, PLC, or computer. It is responsible for the analysis and processing of the measured data, decision-making, and sending of the proper action signals to the output ports (e.g., alarming signals such as message, email, buzzer, light, and so on).

### 1.3. Wireless Communication Technology

To enable mobile systems, suitable communications technologies have to be integrated for the transfer of the measurement data. At least one communication bus is required for the transfer of the acquired data to the server, monitoring, or processing devices. Typical wireless communication principles which can be found in gas sensors are blue-tooth, Wi-Fi, ZigBee, GSM. 

### 1.4. Power Supply

Due to the requirement of mobility, batteries are usually selected as the power supply for the selected systems in the presented work. Besides the direct use of a battery also indirect use is possible when the system is powered from the host robot, PC, or other additional devices. 

### 1.5. Monitoring and Alarm

Monitoring and alarm are the final stages after detecting abnormal situations. As the presented work investigates detecting and alarming system, all the included systems should have this part to be eligible for inclusion. The monitoring and alarming can be implemented in several methods such as on-screen show alarm, SMS message, Email, light signals, buzzer, hybrid, and so on. 

## 2. Materials and Methods

In this work, we focus on mobile detection and alarming systems for hazardous and toxic gases and vapors that can be used especially in laboratories and chemical companies. This work aims to discuss the available technologies for the mobile system and possible solutions for active personal safety in such places. 

### 2.1. Search Strategy

The presented work includes available references until August 2021. Several keywords have been used to import the most relative articles, conference papers, book chapters, and studies such as ‘(hazardous/toxic/poison/noxious) gas (sensor/detector)’, ‘hazardous gases mobile safety system’ and ‘IoT based hazardous gases alarming systems’. The search was initially started at IEEE explore and Scopus database and then expanded to the other listed databases. More than 200 related papers have been imported from IEEE, Scopus, WoS, PubMed, and the ACM library for the period between January 2010 to August 2021 for eligibility. A selection of the most relevant papers was selected to be discussed in the presented work. We focus on several points that help as for inclusion/exclusion criteria. The systematic review was done following PRISMA protocol.

### 2.2. Inclusion and Exclusion Criteria

The term “mobile” refers to the detection and alarming systems, that can be placed and operated stand-alone at any location similar to a stationary unit and can be moved without establishing new infrastructures. Further, we focus on small systems which might be integrated and used as personal worn safety equipment. Moreover, the proposed work focuses on the systems that can measure the concentration of specific hazardous (toxic, poisoned, and noxious) gases even if it is part of the hybrid system (e.g., fire, smoke, personal safety, fall detection, and so on.). General-purpose systems for smoke, fire, or simple air quality index (AQI) detection, without measuring the hazardous components (hazardous gases) in the surrounding environment are not included. The term “alarming” refers to the need to alert, warn or alarm the laboratory personnel of hazardous gases. The alarming functionality can be implemented using sound, buzzer, lights, SMS messages, E-mail, or at least an online concentration level monitoring. Accordingly, the inclusion and exclusion criteria are:

- Inclusion Criteria
  - real-time system, small size
  - hazards or toxic gases sensors included
  - mobile system (movable, battery-powered, wireless technologies)
  - includes monitoring and alarming functions
- Exclusion Criteria
  - fixed/stationary system
  - simulation, algorithms, sensor testing, and manufacturing research’s
  - systems for general smoke, fire, and air quality without measuring specific hazardous and toxic gases levels

**Figure 3:**
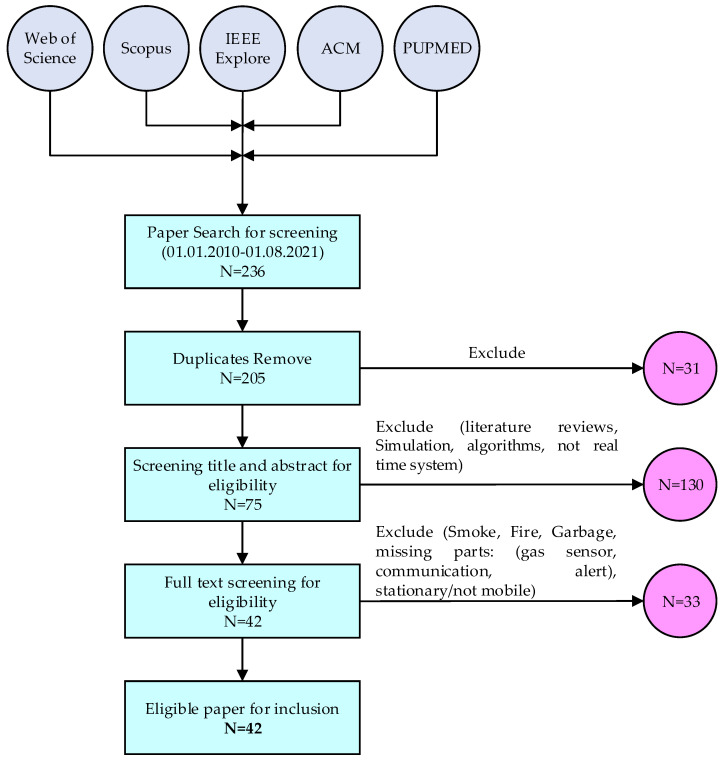
Flowchart of the papers selection process.

Based on the used inclusion criteria, 42 papers were included that met the aim of the proposed study. Figure 3. illustrates the flowchart of the paper’s selection process. 

## 3. Mobile Detecting and Alarming System

Hazardous gases and compounds are widely used in many industrial applications. The protection of employees from harmful gases and vapors requires constant control and monitoring of the concentration of critical substances. Monitoring personal exposure is becoming more and more important here. In addition, indoor air monitoring also plays an important role in increasingly automated environments. To achieve the greatest possible flexibility here, suitable monitoring systems must be mobile and flexible in use, in contrast to the previously dominant permanently installed monitoring devices e.g., hydrogen or carbon monoxide gas emissions. Many researchers worldwide develop detecting and alarming system for hazardous and toxic gases to provide a fast response to accidents and to increase the safety conditions of the employees. Several sophisticated types of gas sensors and detectors have been combined with different communication technologies and processing devices to create a compact solution. The presented review paper does not pursue the aim of a complete evaluation of all available and reported systems. We will include most of the prominent systems that have the main conditions for inclusion, as it is declared in the previous section. We have focused on compact mobile systems that provide a wireless, Internet of things (IoT) based monitoring and alarming for targeted gases data which is more integrated into Industry 4.0. Table 1 summarizes the main parameters of the communication technologies used in the targeted systems [7].

### 3.1. The Used Communication Technologies

Wi-Fi communication technology has been widely used in 46% of the selected papers. Velladurai et al. [23], developed a human safety system for hazardous gases detection and alerting. The system consists of a PIC16F887 microcontroller (Microchip Technology Inc., Arizona, United States) combined with several gas sensors such as MQ-4, MQ-136, and MQ-7 (Winsen Electronics Technology, Zhengzhou, China) to sense the concentration level of H_2_S, CO, and CH_4_ gases. Both sensors are metal oxide semiconductors; the change in the conductivity of the semiconductor material depending on the gas type is used for the gas quantification. The concentration of the toxic gases has been continuously monitored by a mobile application using a Wi-Fi module. The measured gas concentration data are displayed on an LCD screen. When the gas depending threshold is exceeded, an alarm will be triggered and the system will generate and send a warning SMS message to an authorized person. Details on the type of the used power supply and power consumption data are missing. 

Sanger et al. [24], designed a sensor-based system for detecting methane (CH_4_), hydrogen sulfide (H_2_S), and ammonia (NH_3_). The system uses a basic Arduino Uno microcontroller for recording the sensor data, combined with the Node MCU ESP8266 for wireless communication (Espressif Systems, Shanghai, China). The MQ-136, MQ-137 (Winsen Electronics Technology, Zhengzhou, China), and TGS-2611 (Figaro Engineering Inc., Osaka, Japan) gas sensors are used for quantitative gas detection. The acquired data are transmitted from the sensor node to the user monitoring device/database via the ESP8266 Wi-Fi module using the internet network. The system includes a PHP-based web server which enables the visualization of the data on a laptop or mobile device and also stores the acquired data in a database. The authors did not clearly explain the advantage of using two microcontrollers (Arduino UNO + Node MCU ESP8266) at the same time when the Node MCU may be enough for data acquisition, processing, and transmission. Similar approaches have been used on [25,26,27,28,29,30,31,32,33,34,35,36,37].

ZigBee/XBee communication technologies have been used in 17% of the selected research. Cheung et al. developed a real-time safety monitoring system for hazardous gases [38]. The building information modeling (BIM) and the wireless sensor network (WSN) were integrated using Visual Studio and C# application to detect and alarm hazardous gases. The system consists of several compact portable/stationary sensor nodes which enable gas detection. Each sensor node has an MQ-2 gas sensor and Microsoft ARM processor (Gadgeteer (Microsoft, Albuquerque, NM, USA)) combined with a ZigBee-based wireless communication module (CC2530, Texas Instruments, Sherman, TX, USA). The sensor node has been powered by six 18650/3.6 V lithium-ion batteries, which were enough for node operation of 5–6 days. The information from the sensor node can be received by a “Coordinator” connected to the PC of the safety manager. The coordinator is a host which collects all sensor data and sends them to the monitoring PC. The system’s real-time testing results provided the exact location beside the alert and the detected gas data of any abnormal event, and also controlled the ventilation system of the place to reduce the level of detected gas concentration. Eamsa-ard et al. [39], proposed a wearable system for humanoid robots to detect several hazardous gases. The system uses an array of nine gas sensors combined with a LilyPad Arduino 328 microcontroller (Microchip Technology Inc., Chandler, AZ, USA) for data processing. The acquired data can be transferred via an XBee transmitter to a PC with an XBee receiver. The system has been tested with different VOCs such as acetone, ethanol, acetic acid, ammonia, trimethylamine, methanol, dimethylamine, and dipropyl amine. The results indicate the potential of the smart textile fabric as a consumer point-of-care wearable to track the health status and assist in detecting toxic gas leakage in the environment. Other ZigBee/XBee-based WSN has been proposed in [40,41,42,43,44].

The classic Bluetooth and the Bluetooth low energy (BLE) communication technologies have been used in 11% of the targeted research. Choi et al. [45], developed a reconfigurable resolution hazardous multi-gas detection prototype. It consists of a microcontroller with several sensors such as MQ-7, MQ-8, and GSNT11 (Ogam Technology Co., Kwangju, Korea) built in a single board. A Bluetooth wireless module has been used for the prototype communication with a smartphone for data post-processing and viewing. The prototype has been tested with several gases such as CO, SO_2_, C_6_H_6_, CO_2_, NO, N_2_ and CH_4_; the detected gas concentrations are displayed on the smartphone screen. Information on the power supply and the operation time of the system is not described. Heng et al. [46], proposed a hazardous chemicals detection and warning system based on an environmental mobile device. The system uses an Arduino UNO microcontroller with CO and NO_2_ gas sensors. The data transition is realized using a Bluetooth transmitter and receiver. The received data are processed and visualized at the android application as well as a web application that allows the data monitoring on a PC, mobile phone, or tablet. A quad-rotor Unmanned aerial vehicle for hazardous gas detection has been reported by Shi et al. [47]. The system uses an ARM-based microcontroller combined with a Bluetooth 4 communication module to send the acquired data to a mobile application for data monitoring. They used a basic MQ-2 gas sensor for the detection of C_2_H_5_OH, CO, and H_2_. Information on the power supply and the operation time of the system are not described. 

Another group of researchers used Global System for Mobile (GSM) as a communication medium, which represents 22% of the included researches. Jualayba et al. [48], proposed a GSM-based notification hazardous gas detection system. Three basic gas sensors MQ-2, MQ-5, and MQ-8 were tested for hydrogen, LPG, and methane gases. The system is controlled by an Arduino UNO microcontroller which receives and processes the input data from the different gas sensors and sends the appropriate responses via three types of alarming and notification functions which are light signals (three-color based on danger level), buzzer, and SMS notification for the operator. The system has a limit detection distance of approximately 30 inches (76.2 cm). The paper did not include information about the power consumption and the type of power supply. Similar procedures have been used on [49,50,51,52,53]. 

### 3.2. Systems with Robotics Integration 

Robotics have been used in several targeted research due to it is flexibility and mobility through dangerous and unsafe environments. Chang et al. [32], proposed a two-wheel robot-based multipurpose monitoring system that included a hazardous gas detection and alarming part. The system is managed by a Linux operating system based on Raspberry Pi 3 which includes a 1.4 GHz CPU, Bluetooth, and Wi-Fi functionality. The system can deal with several toxic gases and chemical vapors such as PM2.5, CO_2_, LPG, CO, NH_3_, NO_2_, C_3_H_8_, C_4_H_10_, CH_4_, H_2_, and C_2_H_5_OH. The system updates the gas concentration levels using Wi-Fi/4G wireless network and displays them on a smartphone application. The system did not face remote-control restrictions with the control person via the Wi-Fi network. Palacín et al. [54], proposed the use of a humanoid robot as hazardous gases leak detector for safety purposes. An array of 16 MOX gas sensors of 4 types (4* TGS 2611, 4* TGS 2620, 4* TGS 2600, 4* TGS 2602(Figaro Engineering Inc., Osaka, Japan)) has been used for the detection of some VOCs such as acetone and ethanol. The sensors array has been hosted by the assistance personal robot APR-02 which was provided beside the mobility function, the main processor STM32F407VGT6 for data processing, the power supply for the sensor array, and the wireless data transfer to the control station via a Wi-Fi communication module. The transmitted data from the robot can be viewed on a PC/Laptop. The system has been tested continuously for gas measurements and the tests show the system’s ability to detect the gas leakage not only in the direct place but also in the contiguous room with closed doors when there was a small airflow passing under the doors. Fan et al. [55], propose an emergency response system for hazardous gases and chemicals using a mobile robot with an electronic nose. The system sensing hazardous gases using a prototype sensors array called UWAR nose consists of three different material MOX sensors (tin oxide (SnO_2_), nickel oxide (NiO), and tungsten oxide (WO_3_)) and they have been used to measure the concentration of C_2_H_5_OH, C_3_H_6_O, CO, and NO_2_ gases. The electronic noise has been hosted by SmokeBot tank robot (www.smokebot.eu, accessed on 30 November 2021) which is designed specially to operate in dangerous environments, and it is designed as part of an experimental robotic platform called “Taurob tracker” that allow easy and quick adaptation of several sensors, detectors, and devices with the host robot. The robot transmits the sensor data via a Wi-Fi module to the control station, where the received data can be monitored using a PC/laptop system. Barber et al. [56], proposed a gas leakage inspection system for industrial environments. An infrared (IR) imaging technique using an IR camera combined with an interference filter and an IR source has been used for CH_4_ and CO gases detection. The gas inspection system has been fixed on a TURTLEBOT personal mobile robot (Clearpath Robotics Inc., Kitchener, ON, Canada) which has a laptop attached to it for covering the processing, communication, and navigation tasks and also provide the gas inspection system by the required power for a maximum of two hours continuous operating time. The gas inspection results can be viewed on the control station using a PC/Laptop system. Another Robotic-based system approach has been used in [57,58,59,60,61]. 

### 3.3. Systems Hosted by Drones/Quadcopters 

Another group of researchers uses drones and quadcopters in gas detection and alarming implementation. Gallego et al. [62], presented a micro drone-based mobile hazardous gas detecting and alarming system. The system is designed to operate in dangerous, hazardous, and possible toxic gas leakages/emissions areas. An unmanned Aerial Vehicle holds the system components. It consists of the NXP JN5148 microcontroller (NXP Semiconductors Eindhoven Netherlands) adapted with the SIM-908 module which has GSM/GPRS and GPS functionality that has been required for the location information as well as for wireless communication. The used microcontroller has also a ZigBee wireless network module. The system is powered by three batteries (1.5 V, 2500 mAh, AA-type) connected in series to provide a 4.5 V operation voltage. The system uses MiCS-5121 and MiCS-5525 MOX sensors (SGX Sensortech, Corcelles-Cormondreche, Switzerland) for the detection of CO gas and VOCs. Burgués et al. [63], developed a quadcopter/based hazardous gas source localization and mapping. The gas sensing layer consists of two TGS 8100 (Figaro Engineering Inc., Osaka, Japan) MOX gas sensors used to detect gas sources, and it has been tested for ethanol C_2_H_5_OH leakage detection. The sensor has been hosted by the CrazyFlie 2.0 (Bitcraze AB, Malmö, Sweden) quadcopter which is low-cost and small size (10 × 10 cm) and has an open for integration hardware/software that makes the adding/modifying of system elements easy. The system used a powerful 32-bit Cortex-M4 STM32F405 ARM microcontroller (STMicroelectronics, Geneva, Switzerland) for data processing and driver control. The system used a 240 mAh small battery which can provide power for only 7 min and with a maximum of 15 g of payload. The CrazyFlie 2.0 communicates and exchanges sensors data and system parameters (speed, battery level, position, etc.) with the ground station using a 2.4 GHz RF band. The system information including sensors gas data has been monitored on the ground station using a PC/Laptop. Xiaoyuan et al. [64] presented a solution for the detection of hazardous gases concentrations using a quadrotor. The system gas detection layer uses the low-cost MP-3 Flat Surfaced MOX gas sensor (Winsen Electronics Technology, Zhengzhou, China) for ethanol C_2_H_5_OH detection with a measurement range of 10–1000 ppm. The sensor has been adapted on a small 60 × 60 × 31.5 cm quadrotor with 4 Omni-directional wheels, which make it fly or move on the ground based on the targeted unapproachable environment. The system communicates with the ground control station using Wi-Fi communication protocol. Shi et al. [47]. Developed a hazardous gas detection system based on a quad-rotor unmanned aerial vehicle (UAV) drone. The gas sensing layer of the system used the MQ-2 gas sensor for the detection of CO, C_2_H_5_OH, and H_2_. The system parameters control and the sensor’s data processing have been realized using the 32-bit Cortex-M3 STM32F103VET6 ARM microcontroller (STMicroelectronics, Geneva, Switzerland). The UAV drone communicates with the ground control station using a Bluetooth 4 communication module. The already processed gas sensor data will be received by a host computer for data monitoring, and the host computer will send the same data to targeted users using a mobile application.

## 4. Results

We investigated the included systems in several factors. The first factor is the type of used gas sensors. Most of the systems in this study have multiple gas sensors, which are important to extend the ability to detect a variety of hazardous and toxic gases. The review shows that the MOX technology has been used in 31/42 systems, whereas electrochemical gas sensors were used in 9 systems. Other types include catalytic, photoionization, and optical gas sensors that have been used in 12 systems. A majority of 25/42 systems use a multi-gas sensor approach with 2 or more sensors. Table 2 shows a selection of currently available gas sensors from several known vendors that can be used in new system design.

**Figure 4:**
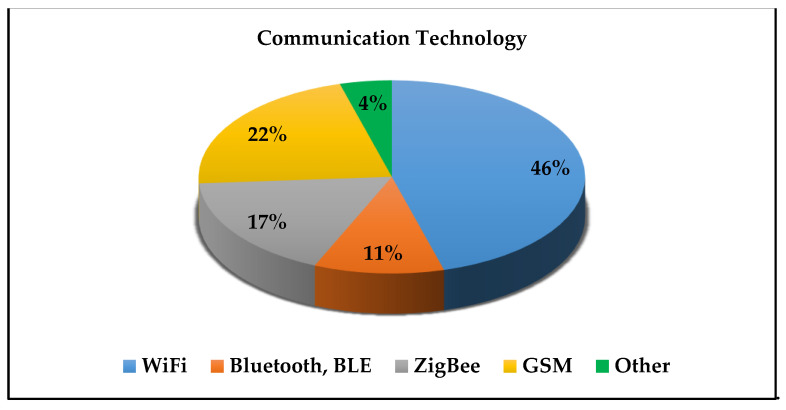
Distribution of the used communication technologies in the presented study.

For operator safety, most of the targeted systems are designed to be used far from the control station or the operator person, thus the selection of the communication modules is one of the important factors in the system performance. The communications requirement for indoor/outdoor, coverage distance, power consumption, and cost should be taken into the consideration at the selection phase of system development. Figure 4. shows the distribution of the used communication technologies in mobile hazardous gas detecting and alarming systems. 

**Figure 5:**
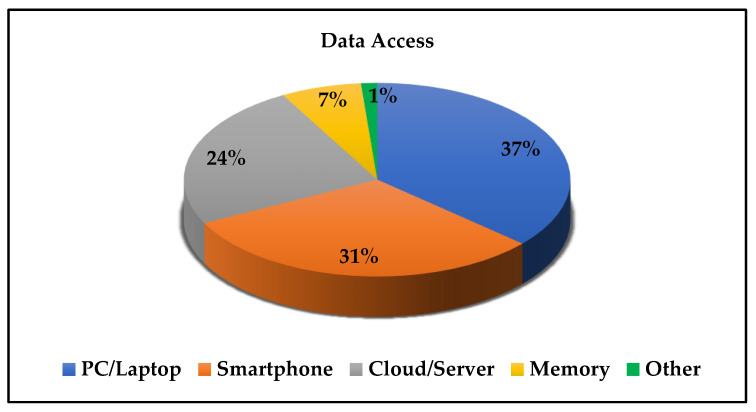
Distribution of the used data access technologies.

Several methods are used to access and/or monitor the targeted gases concentration levels and/or to alert the targeted users (see Figure 5). The majority of solutions use a PC/Laptop or Smartphone connection (37% and 31% respectively). 24% of the reviewed applications use direct cloud/server communication for data transfer. Only 7% store the data directly in internal memory, 1% use other technologies such as monitoring the data via small LCD on handheld, and portable units.

**Figure 6:**
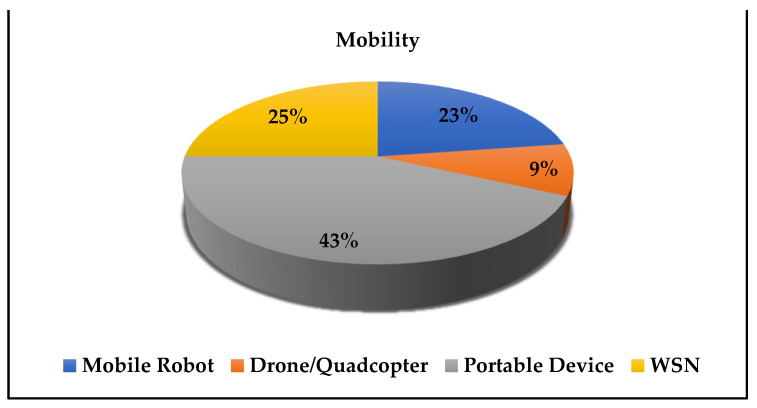
Distribution of the mobility type of the proposed systems.

The main goal of this study is to investigate mobile systems. The word mobile here is not limited to portable systems, but also includes compact standalone units that can be transferred to another location without establishing new infrastructures. Such portable devices represent the majority of currently available mobile systems (43%). The second-largest group is solutions based on wireless sensor networks (25%). In 23% of the selected systems, the mobile robots have been equipped with gas sensors, followed by 9% in drones and quadcopters (see Figure 6).

We summarized the commercial name, type of sensing elements, the parameter measured, used communication modules, the main processing unit, the method of data access, and the mobility of the included systems in Table 3. 

## 5. Discussion

The data in Table 3. clearly show the current directions of developing and improving the hazardous gases detection and alarming systems. The following subsection will discuss the important main direction in targeted systems developments.

### 5.1. Hybrid/Multi-Sensor Systems

One of the important factors in developing toxic and harmful gases detection is the use of hybrid/multi detection sensors. This allows a parallel detection procedure for the target gases. Each vendor of gas sensors may use a different manufacturing method even if they use the same sensor technologies such as MOC, electrochemical, optical, and so on. The importance of this step can be summarized in the following important advantages: 

The system detection results of using hybrid/multi-sensors will be more authenticated than the result of using a single sensor, where the occurrence of false-positive/negative errors is more possible. The hybrid sensing elements may use different manufacturing/detection technologies/methodologies, which enhance the performance and increase the reliability and sobriety of the system [65,66,67,68,69,70]. For example, instead of using one MOX sensor for detecting CO_2_ gas, we can use one MOX and one optical sensor in parallel to detect the CO_2_ and if both of them have the same range of responses this means we get a very reputed measurement in comparison to a single sensor response. 

The use of several sensors will increase the number of hazardous gases that can be detected by the systems, where each additional sensor can detect a different spectrum of toxic/harmful gases. 

The review shows that ≈ 59% of the included systems use more than one sensing element. Systems in [24,25,28,30,31,32,38,54], are good examples for implementing hybrid/multi-sensor detection procedure. 

**Figure 7:**
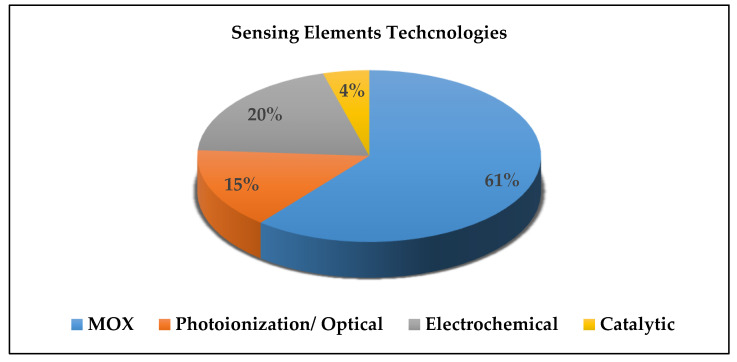
Distribution of the used sensing elements technologies.

Moreover, 58% of the selected system used MOX-based sensing elements, 19% used electrochemical sensing elements, 15% used optical sensing elements, and 4% used catalytic sensing elements. Figure 7. explains the distribution of the used sensing elements technologies. 

### 5.2. Communication Technology

The systems in [27,29,30,40,49,50] are good examples for IoT-based implementation which is one of the important improvements in the ongoing and next generation of hazardous gases detection and alarming systems design [71,72]. As the presented review focus on the mobile systems, we found that the IoT-based systems can achieve the following advantages over implementations:

- More reliable multi-parameter monitoring and alarming
- Better battery-based lifetime and power consumptions
- Multi-Devices communication ability
- More adapted to AI
- Easy adapted with popular used personal smartphone/tablet devices

Several systems in this review use ZigBee communication modules to establish the wireless communication between the sensors node and the monitoring station, which is a good choice for indoor short-range data transferring in WSN. Several systems use the Arduino UNO, Mega, etc., with external Wi-Fi modules for low-cost wireless communication implementation, which can be replaced for example by using the low-cost, smaller, faster, and bigger memory ESP-WROOM-32, WeMos D1 mini, or Node-MCU Arduino-based microcontroller with embedded Wi-Fi module. 

### 5.3. Robotic Based System

Nowadays, robotics is widely used in industrial and laboratory infrastructures. Humanized and wheeled robots are an excellent choice for implementing detection and alarming tasks in harsh/dangerous environments. The robotic-based systems allow a more flexible motion in different environments. The systems can reach very close to the gas/solvent leakage region. They are less affected by toxic and dangerous gases compared to the human operator and can thus be operated in tough conditions. Most of the mobile robots have been designed with a good communication protocol to communicate wirelessly with the control station for receiving and updating the required tasks and at the same time sending the acquired information, robot location, battery charging level, and so on. The already existing communication module enables the sending of measured gas level data and the alarming signals of the hosted gas sensors unit.

23% of the systems in this review are robotic implementation. The systems in [32,54,55,58,61] are good examples for achieving the advantages of using robotic-based systems. In these systems, several gas sensors have been adapted with robot infrastructures which provide a bigger memory size for gas levels data storage, powerful processor, additional longer lifetime power supply, ready to use wireless communication module, and ready to use robot monitoring application which can be used for gas data monitoring and alarming be-side the classical robot controlling and monitoring tasks. 6/10 of the included robotic-based system use Wi-Fi, 1/10 uses ZigBee, 1/10 uses GSM, and 2/10 did not provide information for the used communication protocol. Likewise, 6/10 systems were stand-alone units that have been hosted by a robotic system, and the remaining 4/10 systems were adapted with robotic components and cannot operate independently. The previous robotic-based examples show that the robotics-based hazardous gases detection and alarming systems reduce the time and efforts of system development by offering most of the vital system parts by the host robot. In addition, the robotic implementation is the closest and most credible form of the word “mobile” especially for indoor applications where the drones/quadcopter offer similar advantages to robots for outdoor applications. 

### 5.4. Data Accessing and Alarming

**Figure 8:**
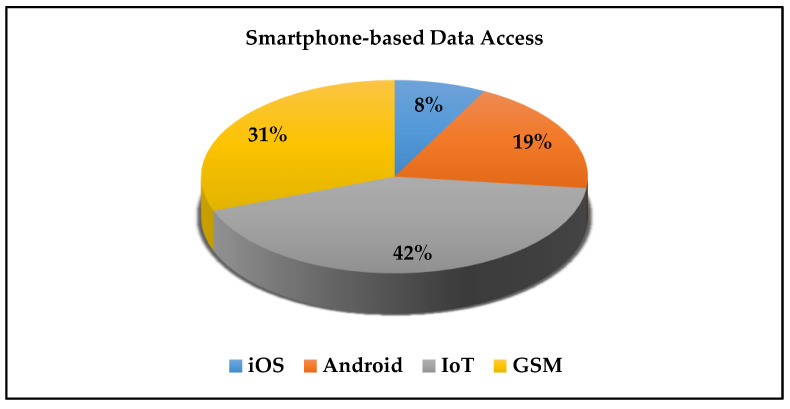
Data access platforms for smartphone-based systems.

The monitoring of the hazardous/toxic gases concentration and alarming in case of critical values represent the second most important part besides the gas detection. The data accessing can be achieved using a cloud server, PC, laptop, tablet, or smartphone. Some systems have more than one data access port. Some systems have on system screen for online data monitoring. All the included systems have a part/unit that deals with the data accessing, monitoring, and alarming. Only 25% of the systems can store the data and make it available in the cloud/database server at any time after direct monitoring, which may be important for future monitoring/processing of the already stored data. Furthermore, some systems such as [29,31,32,35,37,46] allow the users to access the gas data/information from several devices (PC/laptop/Smartphone) which is crucial to alarm the people in the danger domain directly. The systems in [33,40,50] extend the alarm system to an SMS message, Email, buzzer, or light signals beside classical monitor screening. Besides the fact that most people use their smartphones frequently, we can guarantee that most of them hold their alarming devices with them in a harsh environment. The use of the smartphone as one of the data monitoring and alarming platforms makes the alarming goal of the targeted system much practical and applicable and guarantees a fast response from the user side. The review shows that 8/23 smartphone-based systems use the GSM interface for alarming the operators/laboratory personnel by sending SMS messages. This is a very flexible method that does not require a special application in the phone and can be accessed by all types of available mobile phones, even the old generation. Furthermore, 5/23 smartphone-based data accessing and alarming systems used Android application, as well, only 2/23 supporting both android and Apple iOS smartphone applications. 11/23 of the reviewed systems use IoT for data accessing, monitoring, and alarming. The IoT- based system represents ≈ 48% of the included systems. This technology is mainly used in recently published research papers. The use of IoT-based systems makes the data processing, monitoring, and storing more flexible, especially when the sensor data fusion is required or specific algorithms need to be used to calculate gas-related parameters such as air quality index, total volatile organic material index, equivalent CO_2_, and so on. [73,74,75,76]. Figure 8 illustrates the distribution of the data access platforms of the included smartphone-based system. 

## 6. Conclusions

In this review, we investigate hazardous gas detection and alarm system implementation in the past 10 years. We discuss the selected system from different angles/directions such as the used communication modules, sensing elements, data access ports, portability, and alarming, which may be important for the researchers who plan to start designing or developing a new system. Based on the acquired data, we thought that the new researches in this domain should consider the use of IoT-based processors/microcontrollers which can more easily be adapted with deep learning, big data, and artificial intelligent algorithm which is important in the toxic/harmful gas prediction especially for the places that may affect by several kinds of hazardous gases. Furthermore, the hybrid/multi-sensors configuration should be used to avoid the possibility of false-positive alarm and extend the spectrum of hazardous gases that can be detected. 

## Figures and Tables

**Table 1:** Wireless communication technologies are used in hazardous gases mobile detection and alarming systems.

Wireless Technology	Protocol	Coverage Range	Frequency	Data Rate	Power Consumption	Net. Topology
Wi-Fi	IEEE 802.11	∼30–250 m	2.4, 3.7, 5 GHz	>45 Mb/s	High	P2P, Star, Tree
Bluetooth, BLE	IEEE 802.15.1	∼100 m	2.4 GHz	1–3, 1 Mb/s,	Low, Very Low	P2P, Star
LoRa	LPWAN	∼10 Km	868,915 MHz	50 Kb/s	Very Low	Star, Mesh
ZigBee/XBee	IEEE 802.15.4	∼10–100 m	868,915 (MHz), 2.4 (GHz)	250 Kb/s	Medium	P2P, Star, Tree, Mesh
GSM 2G, 4G	GSM	GSM network coverage (>10 km)	933–960 MHz	270 Kb/s, 3.6 Mbit/s	High	TDMA/FDMA

**Table 2:** New in market available gas sensors.

Parameter	ENS160	MiCS-VZ-89TE	SGP40	ZMOD4410	TED110	BME688
Target gases	TVOC, eCO_2_, AQI	TVOC, eCO_2_, AQI	AQI	TVOC, eCO_2_, AQI	TVOC, eCO_2_, AQI	TVOC, eCO_2_, AQI
TVOC range	0–65,000 ppm	0–1000 ppb	0–1000 ppm	0–1000 ppm	0–1000 ppm	-
eCO_2_ range	400–65,000 ppm	400–2000 ppm	-	400–5000 ppm	-	-
“Warm-Up” period	1 min	15 min	<60 s	2 min	-	2 ms
Response time	-	<5 s	<10 s	5 s	10 s	8 s
Refresh Output Frequency	1 MHz	1 Hz	1 Hz	100 kHz	-	182 Hz
IoT devices	Yes	No	Yes	Yes	Yes	Yes
communication	I2C, SPI	I2C	I2C	I2C	I2C	I2C, SPI
AI	No	No	No	Yes	No	Yes
Technology	MOX	MOX	MOX	MOX	MOX, MEMS	MOX, MEMS
Power consumption	-	-	2.6 mA at 3.3 V	1.5 mW	3.9 mW	<0.1 mA
Lifetime	-	-	>10 years	10 years	>5 years	-
Operation voltage	1.7–3.6 V	3.3 V	1.7 to 3.6 V	1.7 to 3.6 V	3.3 V	1.7 to 3.6 V

**Table 3:** Summary of mobile hazardous gases detecting and alarming systems.

Reference No., Year	Used Sensors	Sensor Type	Parameter	Communication Interface	MCU	Data Access	Mobility/Host
[23], 2017	MQ-4, 7, 135, 136	MOX	H_2_S, CO, CH_4_	Wi-Fi, GSM	PIC16F887	Smartphone	Portable Device
[24], 2021	MQ-136, MQ-137, TGS-2611	MOX	CH_4_, H_2_S, NH_3_	Wi-Fi	Arduino Uno	Web/Cloud, PC/Laptop, Smartphone	Portable Device
[25], 2018	Au-TiO_2_, Au-SnO_2_, Au-WO_3_, Au-ZnO	MOX, Catalytic	CH_4_, H_2_, N_2_	Wi-Fi	Node-MCU-ESP8266	Web/Cloud, PC/Laptop	Portable Device
[26], 2019	MQ-2	MOX	LPG	Wi-Fi	Node-MCU ESP8266, Arduino Mega 2560	Smartphone	WSN
[27], 2017	MQ-7, 135	MOX	CO, CO_2_, SO_2_, NO_2_	Wi-Fi	Raspberry pi 3, Nucleo F401RE	Web/Cloud, Smartphone	Portable Device
[28], 2018	Sharp-DN7C3CA006, Alphasense-CO-B4, OX-B431	Optical, MOX	CO_2_, O_3_, NO_2_	Wi-Fi	Teensy 3.2, Arduino-ATmega32u4,	Web/Cloud	WSN
[29], 2018	SnO_2_, ZnO	Electrochemical, Catalytic	H_2_S, CO	Wi-Fi/RF, BLE	ESP-WROOM-32	Web/Cloud, PC/Laptop, Smartphone	WSN
[30], 2020	MQ-135	MOX	CO_2_	Wi-Fi	Arduino Uno, Raspberry Pi 3	PC/Laptop	Portable Device
[31], 2019	TGS2620, TGS2603, TGS2600	MOX	C_2_H_5_OH	Wi-Fi	Arduino Uno	Web/Cloud, PC/Laptop, Smartphone	Robot
[32], 2021	MQ-2, Grove-MICS6814, CO_2_ gas sensor	MOX	CO_2_, LPG, CO, NH_3_, NO_2_, C_3_H_8_, C_4_H_10_, CH_4_, H_2_, C_2_H_5_OH	Wi-Fi	Raspberry Pi 3	Web/Cloud, Internal-Memory, Smartphone	Robot
[33], 2021	ME2-O_2_, MQ-4,7,136, MICS-6814	MOX, Electrochemical	CH_4_, CO_2_, N_2_, O_2_, H_2_S	Wi-Fi, BLE, GSM	Arduino UNO	Web/Cloud, PC/Laptop, Smartphone	Portable Device
[34], 2019	MQ-2	MOX	LPG	Wi-Fi	Node-MCU ESP8266	Web/Cloud	Portable Device
[35], 2020	MQ-7,9,136	MOX	LPG, CH_4_, CO, H_2_S, C_4_H_10_	Wi-Fi	Atmega 328P	Web/Cloud, Smartphone	Portable Device
[36], 2020	Reagents	Optical	NH_3_, CO	Wi-Fi	Raspberry Pi 3	PC/Laptop, Smartphone	Portable Device
[37], 2020	PID	Photoionization/Optical	CO, CO_2_, VOC	Wi-Fi	STM32F407IG	Web/Cloud, PC/Laptop, Smartphone	WSN, Portable Device
[38], 2018	MQ-2	MOX	CH_4_	ZigBee	Gadgeteer	Web/Cloud, PC/Laptop	WSN
[39], 2018	NA	Electrochemical	NH_3_	ZigBee/XBee	LilyPad Arduino328	PC/Laptop	Portable Device
[40], 2018	4-SO_2_-20, 4-NO_2_-20, OX-A431, INE20-CO2P-NCVSP, 4-CO-500, 4-Cl2-50	Electrochemical	CO_2_, CO, SO_2_, NO_2_, O_3_, Cl_2_	ZigBee/XBee	ATmega1281	Web/Cloud, Internal-Memory, Smartphone, PC/Laptop	WSN
[41], 2016	Figaro’s TGS4161, KE-25	Electrochemical	CO_2_, CO, O_2_, NO_2_	ZigBee	ATmega1281	Web/Cloud, PC/Laptop	WSN
[42], 2014	Dr¨ager X-am 5000	Electrochemical	NA	ZigBee	Texas Instruments CC2530-CC2591EM	PC/Laptop	WSN
[43], 2011	Figaro’s TGS4161, KE-50	Electrochemical	CO_2_, O_2_	ZigBee	PIC18LF4620	Web/Cloud, PC/Laptop	WSN
[44], 2016	MQ-2	MOX	LPG/CNG	ZigBee	Atmega 328	Web/Cloud, PC/Laptop, Smartphone	WSN
[45], 2018	MQ-7, GSNT11, MQ-5, MQ-8,	Semiconductor, MOX	CO, H_2_, NO, CH_4_, C_6_H_6_, SO_2_	Bluetooth	NA	Smartphone	Portable Device
[46], 2012	NA	MOX	CO, NO_2_	Bluetooth	Arduino UNO	Web/Cloud, PC/Laptop, Smartphone	Portable Device
[47], 2016	MQ-2	MOX	C_2_H_5_OH, CO, CH_4_, H_2_	Bluetooth	STM32F1	PC/Laptop, Smartphone	Drone/Quad-copter
[48], 2018	MQ-2,5,8	MOX	LPG	GSM	Arduino UNO	Smartphone	Portable Device
[49], 2017	MQ-2	MOX	LPG, C_2_H_5_OH, CO, CH_4_, H_2_	GSM	Arduino UNO	PC/Laptop, Smartphone	Portable Device
[50], 2019	MQ-4,7	MOX	CO, CH_4_	GSM	Arduino UNO	PC/Laptop, Smartphone	Portable Device
[51], 2019	MQ-5	MOX	LPG	GSM	Arduino UNO	Smartphone	Portable Device
[52], 2016	PID-AH	Photoionization/Optical	H_2_S, VOC	GSM	ARM-9	Web/Cloud	WSN
[53], 2016	MQ-5	MOX	LPG	GSM	PIC18F	Smartphone	Portable Device
[54], 2019	FIGARO TGS 2600, 2602, 2611, 2620	MOX	C_2_H_5_OH, C_3_H_6_O	Wi-Fi/RF	STM32F407VGT6	PC/Laptop	Robot
[55], 2018	UWAR nose	MEMS, MOX, Optical, Electrochemical	C_2_H_5_OH, C_3_H_6_O	Wi-Fi/RF	STM32	PC/Laptop	Robot
[56], 2015	Infrared	Optical	CH_4_, CO_2_	NA	TurtleBot	PC/Laptop,	Robot
[57], 2015	MultiRAE Lite	Electrochemical, Optical, MOX	CH_4_, CO	Wi-Fi/RF	PowerBot	PC/Laptop	Robot
[58], 2020	MQ-5, MQ-135	MOX	LPG, CO_2_, C_2_H_5_OH	ZigBee	NA	Other	Robot
[59], 2011	Infrared	Optical	CH_4_, CO	NA	NA	PC/Laptop, Internal-Memory	Robot
[60], 2019	MQ-2, MQ-3	MOX	C_2_H_5_OH, CO	GSM	Arduino UNO	PC/Laptop	Robot
[61], 2019	MICS2614, MICS5524, MICS5914, MICS2714, MICS4514	MOX, Optical	C_2_H_5_OH, C_3_H_8_O, C_3_H_6_O	Wi-Fi	Teensy 3.6	Internal-Memory, PC/Laptop	Portable Device, Robot
[62], 2015	MiCS-5121, MiCS-5525	MOX	CO, HC, VOC	GSM, RF	Jennic JN5148	Internal-Memory, Smartphone	Drone/Quadcopter
[63], 2019	TGS 8100	MOX	C_2_H_5_OH	Wi-Fi/RF	STM32F405	PC/Laptop, Smartphone	Drone/Quadcopter
[64], 2016	MP-3 Planar	MOX	C_2_H_5_OH	Wi-Fi	NA	Other	Drone/Quadcopter

## Data Availability

Not applicable.
